# Determination and pharmacokinetics study of oxyclozanide suspension in cattle by LC-MS/MS

**DOI:** 10.1186/s12917-019-1963-0

**Published:** 2019-06-24

**Authors:** Jili Zhang, Yubin Bai, Bing Li, Xuzheng Zhou, Hongfei Si, Jiyu Zhang

**Affiliations:** 1 Key Laboratory of Veterinary Pharmaceutical Development, Lanzhou Institute of Husbandry and Pharmaceutical Sciences, Chinese Academy of Agricultural Sciences, Ministry of Agriculture, Lanzhou, Gansu Province 730050 People’s Republic of China; 2 Key Laboratory of New Animal Drug Project of Gansu Province, Lanzhou, Gansu Province People’s Republic of China; 3 Lanzhou Institute of Husbandry and Pharmaceutical Sciences, Chinese Academy of Agricultural Sciences, Lanzhou, Gansu Province People’s Republic of China

**Keywords:** Oxyclozanide, Niclosamide, LC-MS/MS, Cattle plasma, Pharmacokinetics

## Abstract

**Background:**

Oxyclozanide is an anthelmintic drug that is widely used to treat fasciolosis*.* However, the pharmacokinetics of oxyclozanide in cattle are not yet clearly understood. The present study was designed to develop a sensitive method to determine oxyclozanide levels in cattle plasma using high-performance liquid chromatography-tandem mass spectrometry (HPLC-MS/MS) and to study its pharmacokinetics for application in cattle.

**Results:**

A simple and rapid HPLC-MS/MS analytical method was established and validated to quantify oxyclozanide levels in cattle plasma using niclosamide as the internal standard (IS) in negative ion mode. Chromatographic separation of the analytes was achieved using a C_18_ analytical column (75 × 4.6 mm, 2.7 μm) at 30 °C. The mobile phase comprised 0.01% v/v acetic acid (HOAc) diluted in water:acetonitrile (MeCN) (90:10% v/v) and 5 mM ammonium formate in methanol (MeOH):MeCN (75:25, v/v) at a 10:90 ratio (v/v) and was delivered at a flow rate of 0.4 mL min^− 1^. A good linear response across the concentration range of 0.02048–25.600 μg/mL was achieved (r^2^ = 0.994). The method was validated with respect to linearity, matrix effect, accuracy, precision, recovery and stability. The lower limit of quantification (LLOQ) was 0.020 μg/mL, and the extraction recovery was > 98% for oxyclozanide. The inter- and intra-day accuracy and precision of the method showed the relative standard deviation (RSD) less than 10%. The method was successfully applied to an assessment of the pharmacokinetics of oxyclozanide in cattle plasma. In healthy cattle, a single oral dose of an oxyclozanide suspension followed the one-compartment model, with a half-life (T_1/2_) of 64.40 ± 30.18 h, a plasma clearance rate (CL/F) of 11.426 ± 2.442 mL/h/kg, and an average area under the curve (AUC) of 965.608 ± 220.097 h*μg/mL. The peak concentration (C_max_) was 15.870 ± 2.855 μg/mL, which occurred at a peak time (T_max_) = 22.032 ± 3.343 h.

**Conclusions:**

A reliable, accurate HPLC-MS/MS analytical method was established in our study and successful applied to study the pharmacokinetics of oxyclozanide in cattle plasma. These results will be useful for further evaluations of the pharmacokinetic properties of oxyclozanide or for monitoring therapeutic drugs in animals.

## Background

*Fasciola hepatica* is an economically important parasite that causes the neglected tropical disease of ruminants known as ‘fasciolosis’ and affects agriculturally important ruminant animals [1–3]. It has a composite life cycle that involves a snail as an intermediate host before being transferred to definite hosts, namely, humans and other herbivorous mammals, through contaminated water or green vegetables [1, 4]. Fasciolosis is geographically widely dispersed in Africa, Asia, Europe, the Americas and Oceania, as well as some temperate countries and regions, and causes significant financial losses [4, 5]. Currently, control of fasciolosis in animals is achieved through the use of flukicidal drugs. However, due to the complex life cycle of *F. hepatica*, a lack of monitoring of treatment efficiency, and frequent reports of flukicide resistance [6–8], fasciolosis control is difficult. Oxyclozanide (3,3′,5,5′,6 pentachloro-2′-hydroxy salicylanilide) is a salicylanilide anthelmintic drug that mainly acts by uncoupling oxidative phosphorylation [9]. As a broad-spectrum anthelmintic drug, oxyclozanide has been widely used to treat infections caused by *Fasciola gigantica, F. hepatica*, *Paramphistomum leydeni,* and *Calicophoron daubneyi*, as well as other intestinal trematodes and gastrointestinal nematodes [10–13], and has not been reported to induce resistance. It also continues to be an important drug because of its efficacy against triclabendazole-resistant *Fasciola* species [14]. To date, no pharmaceutical formulation of oxyclozanide is available in China. A new pharmaceutical formulation of oxyclozanide suspension has been successfully prepared by Lanzhou Institute of Husbandry and Pharmaceutical Sciences to meet the clinical needs of veterinarians. This oxyclozanide suspension possesses good physical properties, good stability and dispersion, controllable quality and exerts remarkable therapeutic effects on *F. hepatica* infections [15]. However, the pharmacokinetic profile of the oxyclozanide suspension in cattle has never been measured.

In fact, despite the widespread use of oxyclozanide for many years, little information has been published on the pharmacokinetics of oxyclozanide in cattle plasma. Instead, some methods have recently been developed to detect oxyclozanide residues in dairy animals. For example, levels of oxyclozanide residues in milk have been measured using ultra-high-performance liquid chromatography-tandem mass spectrometry (UPLC-MS/MS) and liquid chromatography coupled with tandem mass spectrometry (LC-MS/MS) methods [16–18]. Meanwhile, the stability of oxyclozanide residues in beef during cooking has been analysed using a UPLC-MS/MS method [19]. High-performance liquid chromatography-tandem mass spectrometry (HPLC-MS/MS) has also been applied to assess the levels of oxyclozanide residues in bovine kidney [20]. In addition, an LC method has been developed to determine oxyclozanide levels in pharmaceutical formulations [21]. An HPLC analytical method has been developed to compare the pharmacokinetics of oxyclozanide in goats and sheep [22].

The aim of this study was to establish a sensitive and reliable HPLC-MS/MS method to analyse the pharmacokinetics of oxyclozanide in cattle plasma and study its application through examination of the pharmacokinetics of this drug in cattle.

## Methods

### Reagents and chemicals

Standard oxyclozanide and niclosamide (Internal stander; IS) were provided by Dr. Ehrenstorfer from GmbH (Germany) (batch numbers C15793000 and C15510000, respectively) with a purity > 99%. Analytical grade methanol (MeOH), acetonitrile (MeCN), acetic acid (HOAc), and ammonium formate were purchased from Fisher Chemical (Waltham, MA, USA). Water was purified through a Milli-Q Plus water system (Millipore Corporation, Bedford, MA, USA) before use. The oxyclozanide suspension (active ingredient oxyclozanide, 3.4%; batch number 20170104) was supplied by the Lanzhou Institute of Husbandry and Pharmaceutical Sciences (Lanzhou, China).

### LC/MS/MS analysis

The LC analysis was performed using an Agilent 1200 series instrument (Agilent Technologies, USA) containing two SL bin-pumps, an on-line degasser, a column oven and an autosampler. The analytes were separated using an Agilent Poroshell 120 SB-C_18_ (75 × 4.6 mm, 2.7 μm); mobile phase A (0.01% HOAc in water:MeCN; 90:10 v/v) and mobile phase B (5 mM ammonium formate in MeOH:MeCN (75:25, v/v) were used as the mobile phase at a ratio of 10:90 v/v. The mobile phase was filtered before use to prevent the entry of bubbles or impurities into the system and was delivered at a flow rate of 0.4 mL/min. The sample was injected at a volume of 5 μL at 30 °C.

The mass spectroscopy analysis was performed using a G6410A triple-quadruple tandem mass spectrometer with electrospray ionization (ESI) (Agilent Technologies Inc., Santa Clara, CA, USA) in negative ion mode. The following MS/MS parameters were used: capillary voltage, 4 KV; source temperature, 330 °C; and nitrogen gas flow rate, 11 L/min. The optimized fragmentation voltages for oxyclozanide and niclosamide were 100 V and 90 V, respectively, and the delta electron multiplier voltage was 400 V. Data were collected in multiple reaction monitoring (MRM) mode using [M-H]^−^ ions for oxyclozanide and niclosamide (IS), with collision energies of 30 and 20 eV, respectively. Mass Hunter software (version B.01.04, Agilent Technologies Inc.) was used for system control, data acquisition, and data processing.

### Preparation of standard solutions

For the standard stock solution of oxyclozanide, 100 mg of oxyclozanide was placed into a 50 mL brown volumetric flask, after which methanol was added to produce a stock solution of 2000 μg/mL oxyclozanide. A series of working oxyclozanide solutions was prepared by diluting the standard stock solution with the mobile phase to obtain the following concentrations (μg/mL): 1.024, 16.384, 32.768, 262.144, 655.360, 1024 and 1280.

For the IS solution, 1.000 mg of niclosamide was placed in a 100 mL brown volumetric flask, to which methanol was added to produce a stock solution of 10.00 μg/mL niclosamide. Next, 1 mL of the niclosamide stock solution was mixed with the mobile phase in a 50 mL volumetric flask to produce a solution of 0.200 μg/mL niclosamide. All solutions were stored at 4 °C and brought to room temperature before use.

### Working solutions and sample preparation

Plasma calibration standards with concentrations ranging from 0.02048–25.600 μg/mL were prepared by adding 10 μL of each of the oxyclozanide standard solutions (1.024–1280 μg/mL) and 10 μL of the niclosamide (0.200 μg/mL) IS solution to 500 μL aliquots of blank plasma. Quality control (QC) samples were prepared in the same way at different oxyclozanide concentrations: 0.020 μg/mL (lower limit of quantification, LLOQ), 0.02048 μg/mL (QC-low), 12.7612 μg/mL (QC-med), and 25.600 μg/mL (QC-high). The calibration standards and QC samples were applied for method validation in the pharmacokinetic study.

Plasma aliquots (500 μL) were spiked with 10 μL of the niclosamide (0.200 μg/mL) IS solution in centrifuge tubes, after which acetonitrile (1.5 mL) was added. The sample was mixed by vortexing (30 s) and centrifuged at 3000×g for 20 min, and the supernatant was evaporated to dryness at 40 °C with a vacuum concentration system (Rapid Vap® Vertex Evaporator, Labconco, USA). The dry residue was reconstituted in 500 μL of mobile phase and immediately subjected to vortexing for 20 s, after which the solution was filtered through a 0.22 μm Millipore filter and injected into the LC-MS/MS system.

### Method validation

#### Selectivity and matrix effect

Selectivity was examined by comparing the chromatograms of eight different batches of blank cattle plasma with those of corresponding plasma samples spiked with oxyclozanide and the IS to exclude the interfering peaks [23].

The matrix effect was evaluated by comparing the area response of post-extraction blank plasma samples spiked with oxyclozanide at three QC levels with the equivalent concentration standard solutions that were dried directly and reconstituted with the same mobile phase [24].

#### LLOQ and linearity

The LLOQ and the lower limit of detection (LLOD) were determined as the concentrations that produced signal/noise ratioes of 10 and 3, respectively. For the linearity of this method, a calibration curve was generated with plasma standards containing different concentrations of oxyclozanide ranging from 0.02048–25.600 μg/mL. A calibration curve was constructed by plotting the peak area ratio of oxyclozanide/IS (y) vs the nominal concentration of oxyclozanide (x) in the form of y = ax + b; the least square method was used for the linear regression analysis. A coefficient of correlation (r^2^) of at least 0.99 was required to meet the criterion.

#### Accuracy and precision

The precision was determined as the relative standard deviation (RSD) of replicate measurements, and the accuracy was evaluated as the ratio of calculated vs. theoretical concentrations, as previously described [24]. The intra-day accuracy and precision of the HPLC/MS/MS method were determined by analysing QC concentrations (0.02048 μg/mL, 12.7612 μg/mL and 25.600 μg/mL) and the LLOD concentration in six replicates per concentration on the same day. Inter-day accuracy and precision were evaluated by analysing QC concentrations (0.02048 μg/mL, 12.7612 μg/mL and 25.600 μg/mL) and the LLOD concentration in six measurements of each concentration conducted over 3 days [25]. According to the ICH [26], the criterion for precision and accuracy was an RSD ≤ 15% for each concentration, except for the LLOQ (≤ 20%).

#### Recovery and stability

Three concentrations of QC samples (0.02048 μg/mL, 12.7612 μg/mL and 25.600 μg/mL) and a single concentration of IS (0.004 μg/mL) were analysed in six replicates to evaluate the efficiency of oxyclozanide extraction from the bio-matrix. Recovery was determined by comparing the analytical results of the extracted QC samples with the pure standards without extraction.

Stability was assessed by analysing replicates (*n* = 6) of the QC samples at concentrations of 0.02048 μg/mL, 12.7612 μg/mL and 25.600 μg/mL under various the sample storage and processing procedure [27]: (1) the plasma samples were kept at ambient temperature for 24 h; (2) the plasma samples were stored at − 20 °C for 60 days; (3) the plasma samples were kept in the autosampler at 4 °C for 24 h; (4) the plasma samples were determined after three freeze-thaw cycles (25 °C to − 20 °C) [25].

### Pharmacokinetic study

Eight healthy breeding age Simmental cattle of both sexes aged between 1.5 and 3 years and with a mean body weight (BW) of 317 ± 24.46 kg were obtained from a commercial company (Wanhe Livestock Industry Technology Development Co., Ltd., Gansu, China) and acclimated to a standard environmentally controlled animal room (temperature, 25 ± 2 °C; relative humidity of 50% and a 12:12 h light/dark cycle) for 1 week before the experiment. Animals were orally administered oxyclozanide once, and the dose was based on live weight. All experimental procedures were approved and performed in accordance with the Guidelines for the Care and Use of Laboratory Animals of the Lanzhou Institute of Animal Science and Veterinary Pharmaceutics (number: SCXK (Gan) 2014–0002). All cattle were maintained under nearly identical conditions, and adequate water and rations were provided.

The animals received an oral suspension of oxyclozanide at a dose of 10 mg/kg BW. Blood samples (5 mL) were collected by jugular venepuncture at 0 h, 0.5 h, 1 h, 4 h, 8 h, 12 h, 16 h, 18 h, 20 h, 24 h, 28 h, 36 h, 48 h, 72 h, 96 h, 120 h, 168 h and 216 h. After all the blood samples were centrifuged at 3000 rpm for 10 min, plasma samples were collected and immediately stored in a − 20 °C freezer until analysis using LC-MS/MS. All the animals were alive and healthy after the experiment.

Pharmacokinetic parameters were calculated using WinNonlin Professional software version 5.2 (Pharsight, Mountain View, CA, USA). The best pharmacokinetic model was confirmed according to the minimum Akaike Information Criterion (AIC) value principle and utilized for data fitting and parameter estimation [28]. Plasma area under the curve (AUC), plasma clearance rate (CL/F), peak plasma concentration (C_max_), half-life (T_1/2_), and peak time (T _max_) are presented as means ± SD.

## Results

### Mass spectrometry and chromatography

**Fig. 1:**
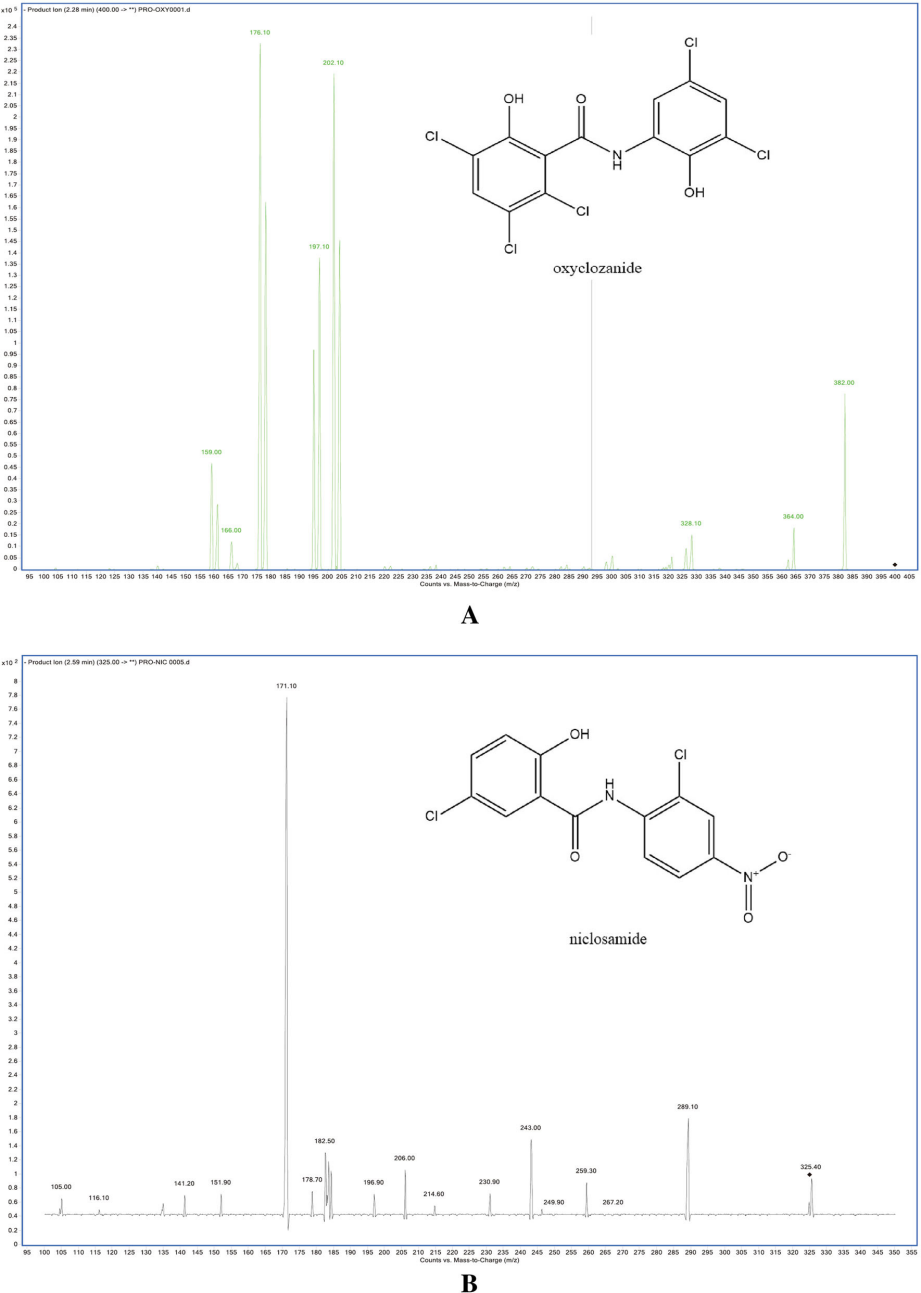
Full-scan product ion spectra of [M-H]^**−**^ ions for oxyclozanide (**a**) and IS (**b**)

In negative ESI mode, oxyclozanide and the IS exhibited good responses. The full-scan ion spectra indicated that the most abundant ions observed for oxyclozanide were [M-H]^−^ ions at m/z 401.46 and m/z 327.12 for the IS. The quantification of oxyclozanide was carried out with MRM mode for high selectivity and sensitivity of acquisition data. The ion transitions of MRM were selected as m/z 401.46 to 176.2 for oxyclozanide and m/z 327.12 to 171.1 for the IS (Fig. 1).

**Fig. 2:**
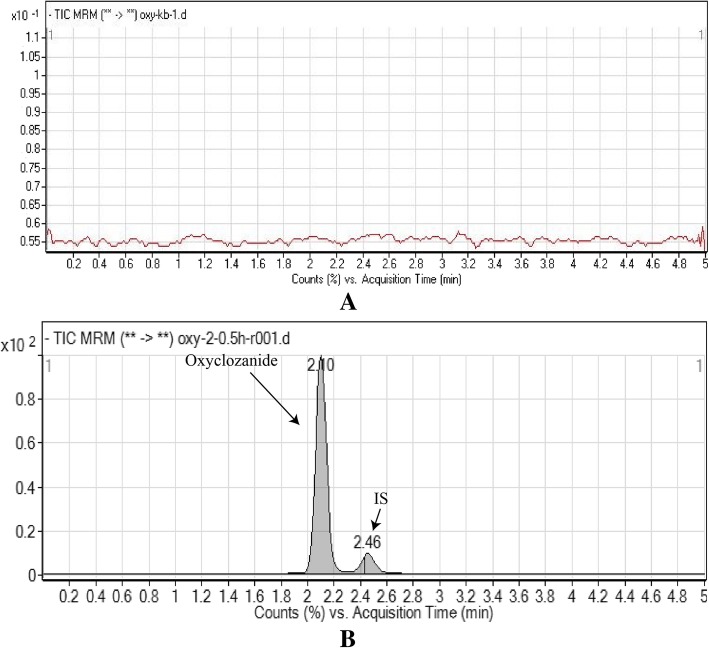
Chromatograms of plasma from cattle. Blank (**a**), oxyclozanide (**b**, left panel) and IS (**b**, right panel)

The analysis was conducted with mobile phase A, 0.01% HOAc in water:MeCN (90:10, v/v), and mobile phase B, 5 mM ammonium formate in MeOH:MeCN (75:25, v/v), at a ratio of 10:90 v/v for mobile phases A and B. A 75 mm column subjected to isocratic elution of the mobile phase for 5 min at a flow rate of 0.4 mL/min was used for chromatographic separation. Under optimized LC and MS conditions, oxyclozanide and the IS were separated with retention times of 2.10 min and 2.46 min, respectively, and endogenous substances in the plasma did not interfere with analyte detection. Figure 2 shows the chromatograms of untreated plasma and plasma containing oxyclozanide and the IS at 5 min after oral administration.

### Method validation

#### Selectivity and matrix effect

The specificity of the method was assessed by analysing each blank plasma sample from eight different cattle using the aforementioned chromatographic conditions. None of the samples exhibited endogenous substance-mediated interference in the retention time of oxyclozanide or the IS.

In an evaluation of the effect of the plasma matrix on oxyclozanide and IS levels in eight diverse blank cattle plasma samples, the mean matrix effect on oxyclozanide was 96.3 ± 2.1%, while the matrix effect on the IS was 95.7 ± 1.6%.

#### LLOQ and linearity

**Fig. 3:**
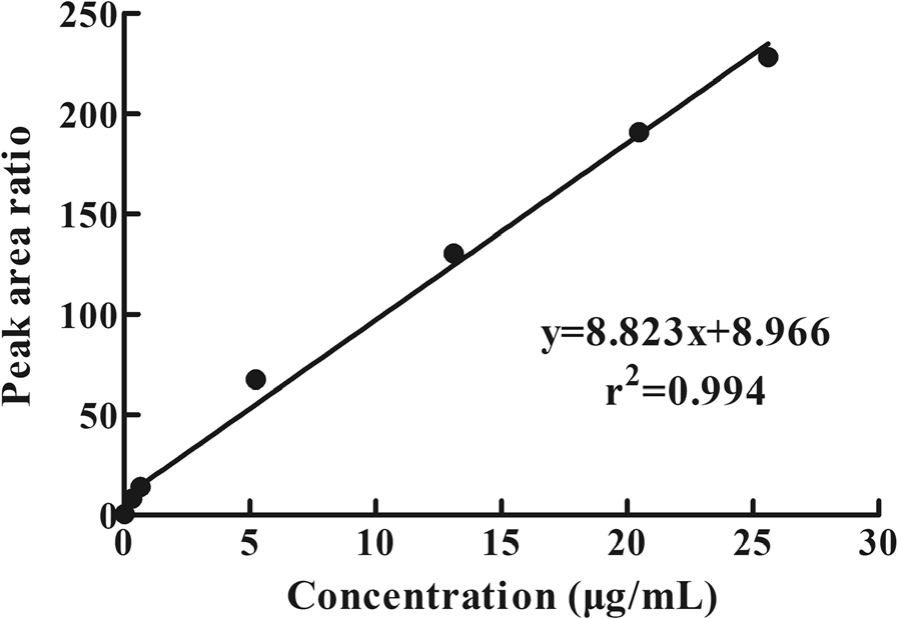
Calibration curve

The LLOQ and LLOD of oxyclozanide were 0.020 μg/mL and 0.010 μg/mL, respectively. The calibration curve for oxyclozanide was linear over the concentration range of 0.02048–25.600 μg/mL according to the results of a weighted (1/x^2^) least-square linear regression analysis. The calibration curves are shown in Fig. 3. The extrapolated equation of the calibration curve for oxyclozanide was y = 8.823x + 8.966 (r^2^ = 0.994) for concentrations ranging from 0.02048–25.600 μg/mL.

#### Accuracy and precision

The results of the analyses of the intra- and inter-day precision and accuracy of the QC samples (0.02048 μg/mL, 12.7612 μg/mL and 25.600 μg/mL) and LLOD (0.020 μg/mL) samples are presented in Table 1. The intra-day accuracy ranged from 97.2 to 126.7%, and the inter-day accuracy ranged from 97.4 to 123.6%. The intra- and inter-day precisions were 0.2–5.1% and 0.5–4.2%, respectively.

**Table 1:** Intra- and inter-day precision and accuracy of oxyclozanide in cattle plasma

Concentration(μg/mL)	Intra-day precision and accuracy (*n* = 6)		Inter-day precision and accuracy (*n* = 18)	
	Accuracy (%) ± SD	RSD (%)	Accuracy (%) ± SD	RSD (%)
0.020	112.8 ± 5.7	5.1	109.5 ± 4.6	4.2
0.02048	126.7 ± 4.2	3.3	123.6 ± 3.7	3.0
12.7612	105.0 ± 0.2	0.2	105.4 ± 0.9	0.9
25.600	97.2 ± 0.3	0.3	97.4 ± 0.5	0.5

#### Recovery

Table 2 displays the recovery of oxyclozanide and IS extracted from the plasma matrix. The mean extraction recoveries of oxyclozanide at QC concentrations of 0.02048 μg/mL, 12.7612 μg/mL and 25.600 μg/mL were 100.8 ± 3.2%, 106.0 ± 0.8%, and 98.1 ± 1.9%, respectively. The mean extraction recovery of the IS was 98.2 ± 1.7%.

**Table 2:** Recovery of oxyclozanide (*n* = 6) from cattle plasma

Concentration (μg/mL)	Recovery (%, *n* = 6)	
	Mean (%) ± SD	RSD (%)
Oxyclozanide		
0.02048	100.8 ± 3.2	3.2
12.7612	106.0 ± 0.8	0.8
25.600	98.1 ± 1.9	2.2
IS		
0.004	98.2 ± 1.7	1.7

#### Stability

The results of stability for oxyclozanide under different storage conditions was shown in Table 3. The accuracy was 90.8–102.2% and precision (RSD%) was 1.7–3.1%, indicating that oxyclozanide was fairly stable under all experimental conditions.

**Table 3:** Stability of oxyclozanide in cattle plasma samples under various conditions (*n* = 6)

Storage conditions	Concentration (μg/mL)	Accuracy ± SD (%)	RSD (%)
Ambient temperature for 24 h	0.02048	100.5 ± 2.1	2.1
	12.7612	98.2 ± 1.7	1.7
	25.600	102.2 ± 2.3	2.3
At −20 °C for 60 days	0.02048	94.5 ± 2.2	2.3
	12.7612	95.1 ± 2.8	2.9
	25.600	98.9 ± 1.9	1.9
At 4 °C in the autosampler for 24 h	0.02048	96.6 ± 2.5	2.5
	12.7612	101.4 ± 1.5	2.6
	25.600	98.4 ± 2.7	2.1
3 freeze-thaw cycles	0.02048	90.8 ± 2.7	3.0
	12.7612	94.6 ± 2.5	2.6
	25.600	96.2 ± 3.0	3.1

### Pharmacokinetic studies

**Fig. 4:**
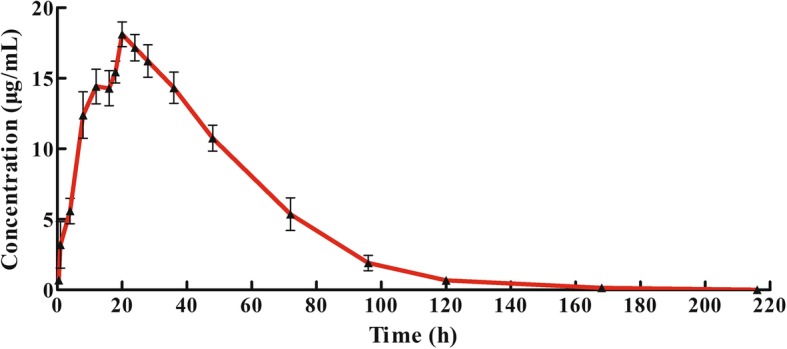
Mean plasma concentration-time profile after the oral administration of 10 mg/kg oxyclozanide to cattle (*n* = 8)

The well-validated method described above was successfully applied to quantify oxyclozanide levels in plasma samples after the oral administration of an oxyclozanide suspension to cattle at a dose of 10 mg/kg. The plasma concentration-time curves for oxyclozanide were adequately fitted using a one-compartment model. The mean plasma concentration vs. time curves for oxyclozanide are shown in Fig. 4. The major pharmacokinetic parameters of oxyclozanide are presented in Table 4. The C_max_, T_max_ and T_1/2_ of oxyclozanide were 15.870 ± 2.855 μg/mL, 22.032 ± 3.343 h, and 64.40 ± 30.18 h, respectively. The AUC_(0-∞)_ for oxyclozanide was 965.608 ± 220.097 h*μg/mL.

**Table 4:** The pharmacokinetic parameters (mean ± SD) of oxyclozanide in cattle following oral administration at a dose of 10 mg/kg (*n* = 8)

Parameters	Mean ± SD
AUC _(0-∞)_ (h*μg/mL)	965.608 ± 220.097
AUC_(0-t)_ (h*μg/mL)	916.534 ± 202.121
K01_HL (h)	14.706 ± 2.509
K10_HL (h)	14.938 ± 2.650
CL/F (mL/h/kg)	11.426 ± 2.442
T_max_ (h)	22.032 ± 3.343
T_1/2_ (h)	64.40 ± 30.18
C_max_ (μg/mL)	15.870 ± 2.855

## Discussion

Full ESI scans were performed in both ESI (±) detection modes to optimize the mass spectrometry parameters for oxyclozanide and the IS. Better MS responses were achieved in ESI (−) mode compared with ESI (+) mode, consistent with previous reports [16, 18, 19]. The choice of the IS is particularly important, as it provides a baseline value for the continuous monitoring of the performance of the chromatograph and mass spectrometer during analysis, and affects the precision and accuracy of the method [29, 30]. Niclosamide is a proper IS due to its structural and chemical similarity to the analyte, its similar retention behaviour to the analyte, as well as be well resolved from the analyte and other peaks. The composition of the mobile phase used in our study was described in previous studies [16, 19, 20]. However, the chromatographic conditions were simplified in our study, and the suitable ratio of the mobile phase was adjusted with equal degrees of elution. Under this chromatographic condition, the retention times were 2.10 min for oxyclozanide and 2.46 min for niclosamide. Retention times and the running time were shorter than in previously published reports [16, 19, 20]. Acetic acid increased the ionization of all tested compounds, whereas the addition of ammonium acetate resulted in higher sensitivity [24]. This composition was deemed suitable for the separation and ionization of oxyclozanide and the IS with good peak shapes and resolution.

In the validation study, the LC-MS/MS method was validated in terms of selectivity, linearity, matrix effect, LLOQ, recovery, accuracy and precision and stability. These variables were assessed according to guidelines established by the US Food and Drug Administration for bioanalytical method validation [31]. A good linear relationship between the ratio of the oxyclozanide concentration and quantitative ion peak area was established. The intra- and inter-day precision and accuracy data were within the acceptable limits, indicating that the method developed in the present study was reliable and reproducible in terms of the quantitative analysis of oxyclozanide levels in cattle plasma. In addition, liquid-liquid extraction with acetonitrile was simple and more efficient than the method described in a previous report [22], and no endogenous substance-mediated interference was observed for the retention time of oxyclozanide or the IS. Furthermore, oxyclozanide was stable in cattle plasma after exposure of the plasma samples to different storage conditions. Thus, a simple and sensitive LC-MS/MS method has been developed and validated in our study.

The pharmacokinetics of oxyclozanide in cattle were assessed using the LC-MS/MS method and the pharmacokinetic properties were obtained in this study. A previously published study reported the pharmacokinetics of oxyclozanide in sheep and goats following the administration of a single oral dose [22]. The T_1/2_ were 21.74 h for sheep and 18.71 h for goats, the AUC_(0–∞)_ values in sheep and goats were 488.70 μg.h/mL and 309.33 μg.h/mL, respectively, and the C_max_ in sheep and goats were 11.01 μg/mL and 6.68 μg/mL, respectively. However, the T_1/2_ of oxyclozanide in cattle plasma reported here was 64.40 h, which is a much longer time than the period in sheep and goats. The AUC_(0–∞)_ (965.608 h*μg/mL) of oxyclozanide in cattle is larger than in sheep and goats, and the C_max_ (15.87 μg/mL) of oxyclozanide is higher than in sheep and goats. The reasons for these differences may be due to the differences in animal species, the selectivity of the analytical methods or the formulation and preparation techniques. However, following the oral administration of an oxyclozanide suspension, the drug is slowly eliminated from cattle plasma, and this property may contribute to the therapeutic effect in vivo. To our knowledge, this study is the first to report the pharmacokinetics of oxyclozanide in cattle, and our results will provide a basis for further research on the administration of oxyclozanide suspensions in animals.

## Conclusions

A simple and sensitive LC-MS/MS method has been completely validated, displayed excellent sensitivity, linearity, precision and accuracy, and was successfully applied to an evaluation of the pharmacokinetics of oxyclozanide in cattle plasma after oral administration. This study is the first to report the pharmacokinetic parameters of oxyclozanide in cattle and will be useful for further evaluations of the pharmacokinetic properties of oxyclozanide or for the monitoring of therapeutic drugs in animals.

## Data Availability

All data generated or analysed during this study are available from the corresponding author on reasonable request.

## Abbreviations

AUC: An average area under the curve
BW: Body weight
C _max_: Peak plasma concentration
CL/F: Plasma clearance rate
ESI: Electrospray ionization
*F. hepatica*: *Fasciola hepatica*
HOAc: Acetic acid
HPLC-MS/MS: High-performance liquid chromatography-tandem mass spectrometry
IS: Internal standard
LC-MS/MS: Liquid chromatography coupled with tandem mass spectrometry
LLOD: Lower limit of detection
LLOQ: The lower limit of quantification
MeCN: Acetonitrile
MeOH: Methanol
MRM: Multiple reaction monitoring
QC: Quality control
RSD: The relative standard deviation
T _max_: Peak time
T_1/2_: Half-life
UPLC-MS/MS: Ultra-high-performance liquid chromatography-tandem mass spectrometry
